# Characteristics of events in which police responded to overdoses: an examination of incident reports in Rhode Island

**DOI:** 10.1186/s12954-022-00698-2

**Published:** 2022-10-18

**Authors:** Alexandria Macmadu, Annajane Yolken, Lisa Frueh, Jai’el R. Toussaint, Roxxanne Newman, Brendan P. Jacka, Alexandra B. Collins, Brandon D. L. Marshall

**Affiliations:** 1 Department of Epidemiology, Brown University School of Public Health, 121 South Main Street, Box G-S-121-2, Providence, RI 02912 USA; 2 Project Weber/RENEW, 640 Broad St, Providence, RI USA; 3 Harvard T.H. Chan School of Public Health, 677 Huntington Avenue, Boston, MA USA; 4 Department of Africana Studies, Brown University Churchill House, 155 Angell Street, Providence, RI USA

**Keywords:** Police, Overdose, Naloxone, Arrests

## Abstract

**Background:**

Narrow or non-existent Good Samaritan Law protections and harsh drug selling statutes in the USA have been shown to deter bystanders from seeking medical assistance for overdoses. Additionally, little is known about the actions that police take when responding to overdose events. The objectives of this study were to assess the prevalence and correlates of naloxone administration by police, as well as to examine overdose events where arrests were made and those in which the person who overdosed was described as combative.

**Methods:**

We analyzed incident reports of police responding to an overdose between September 1, 2019, and August 31, 2020 (i.e., 6 months prior to and during the COVID-19 pandemic), from a city in Rhode Island. We examined characteristics of incidents, as well as individual characteristics of the person who overdosed. Correlates of police naloxone administration were assessed using Wilcoxon rank sum tests and Fisher’s exact tests, and we examined incidents where arrests occurred and incidents in which the person who overdosed was described as combative descriptively.

**Results:**

Among the 211 incidents in which police responded to an overdose during the study period, we found that police administered naloxone in approximately 10% of incidents. In most incidents, police were the last group of first responders to arrive on scene (59%), and most often, naloxone was administered by others (65%). Police were significantly more likely to administer naloxone when they were the first professionals to arrive, when naloxone had not been administered by others, and when the overdose occurred in public or in a vehicle. Arrests at overdose events were rarely reported (1%), and people who overdosed were rarely (1%) documented in incident reports as being ‘combative.’

**Conclusions:**

Considering these findings, ideally, all jurisdictions should have sufficient first responder staffing and resources to ensure a rapid response to overdose events, with police rarely or never dispatched to respond to overdoses. However, until this ideal can be achieved, any available responders should be dispatched concurrently, with police instructed to resume patrol once other professional responders arrive on scene; additionally, warrant searches of persons on scene should be prohibited.

**Supplementary Information:**

The online version contains supplementary material available at 10.1186/s12954-022-00698-2.

## Introduction

Drug overdose deaths in the USA have reached unprecedented levels. Provisional drug overdose death counts data indicate that over 100,000 people died from a drug overdose in the USA in 2021 alone, representing a 15% increase in overdose deaths relative to the prior year [1]. Recent years have represented a continued hyper-exponential increase in overdose deaths that began in 2013 [2], and the overdose epidemic has been further exacerbated by the impact of the ongoing COVID-19 pandemic [3–6]. As overdose deaths continue to surge, naloxone administration by bystanders and first responders has remained a key pillar in US public health infrastructure to prevent overdose deaths.

First responders—including police, fire rescue personnel, and emergency medical services—are routinely dispatched to overdose incidents [7–10]. Given studies showing that police are often among the first to arrive on scene, there has been an increase in the number of overdose response and prevention trainings for police, and officers are often equipped with naloxone [11–14]. However, limited research has documented the role of police in responding to overdose events [7, 8, 15], incidents in which arrests occur [8, 15], or incidents in which the person who overdosed was combative [15, 16]. These previous works also have notable limitations that impact a full assessment of the role of police, including aggregated police and fire attendance data, which prevent the examination of the relative contribution of each agency in overdose reversals [16]. Understanding the actions of police at overdose events is also critical from a racial justice lens, as Black and Indigenous people of color and people who are Latinx are disproportionately affected by police over-surveillance, non-violent drug-related arrests, and drug-related offenses across the USA [17].

Despite knowledge gaps in the role of police during overdose events, extensive prior literature has documented that police attendance at overdose events strongly deters bystanders from seeking emergency services in the event of an overdose [18–26]. While Good Samaritan Laws have been widely implemented to protect both the persons who call 911 in the event of an overdose and the person who has overdosed from arrest [27], many states lack robust Good Samaritan Law protections. Specifically, immunity for arrest for outstanding warrants, probation and parole violations, and immigration consequences are often not included under Good Samaritan Laws [28]. Furthermore, some states have drug-induced homicide laws; these policies seek to establish criminal liability for individuals who furnish or deliver controlled substances to another individual who dies as a result [29], which introduces additional barriers to seeking medical attention in the event of an overdose. For example, in Rhode Island, Kristen’s Law (codified in 2018) permits life sentences for people who sell illegal drugs that result in fatal overdoses [30]. These policies and limitations of existing Good Samaritan Laws—including Rhode Island’s Good Samaritan Law codified in 2016 [31]—thus obfuscate the available legal protections when police respond to overdose incidents. To reduce potential risk of harm, communities of people who use drugs and activist–scholars have called for police attendance at overdose events to end because negative police interactions, fears of arrest, and uncertainty about protections often prevent bystanders from seeking emergency medical services [18–26, 32], which further increases risk of fatal overdose.

In light of current gaps in the literature, the changing legal landscape in Rhode Island with the codification of Kristen’s Law, and persisting racial inequities related to policing in the state [33], we sought to explore characteristics of incidents in which police responded to overdoses by examining incident reports in which police responded to a suspected overdose. Examining these factors in Rhode Island is particularly important given that overdose deaths in the state increased by more than 40% from 2019 to 2021 [34]. The objectives of this study were to assess the prevalence and correlates of naloxone administration by police, as well as to examine incidents in which arrests were made and incidents in which the person who overdosed was described as combative.

## Methods

### Study design and data sources

In November 2020, the study team submitted a public records request to a city in Rhode Island to solicit all incident reports, police logs, and descriptions of incidents of police responding to a suspected overdose between September 1, 2019, and August 31, 2020. We selected this observation period because it captures approximately six months before and after the COVID-19 outbreak was declared a pandemic by the World Health Organization (WHO) on March 11, 2020. A total of 211 redacted police reports (i.e., incident reports) were released to the research team, all in which officers of the local police department responded to a suspected overdose during the 12 months period requested. The city in which this police department resides has a population of greater than 75,000 people and is moderately diverse (i.e., more than half of the population is racially white). The local police department has more than 100 police officers, and all officers in the department are equipped with naloxone and trained in overdose response and prevention.

Each incident report contains seven sections: case, offenses, subjects, arrests, property, vehicles, and narrative. The case section includes the case number (i.e., a unique 12-digit numerical identifier for the incident), the address of the incident, the incident type (i.e., overdose), and the date and time of the incident. The offenses section details the type of offense(s) suspected (e.g., warrant for arrest, drug possession), if any were documented. The subjects section includes basic information about each person from whom police collected information on scene, including the subject type (e.g., victim, other reporting person), name, home address (if available), phone number, race, sex, and date of birth or age. The arrests, property, and vehicles sections catalog relevant incident details if an arrest was made, if property was seized, or if a vehicle was towed from the scene, respectively. The narrative section provides a chronological synopsis of the incident from the perspective of the reporting officer and persons on scene from whom the police collected information. The following identifiers were redacted by city officials from all sections of each incident report: names, addresses (except the city/town, state, and ZIP code), telephone numbers (except the area code), dates of birth (except the year), as well as any other individually-identifiable information. A small proportion (3%) of records were over-redacted, and nonidentifiable information (e.g., ZIP code, age) could not be extracted.

Two members of the study team (AM and AY) developed an initial data extraction form to collect relevant data from incident reports (see Additional file 1: Appendix for the final data extraction form). The initial form was iteratively piloted by the remaining members of the study team (LF, JT, AC, BJ, RN). At this stage, fields in the data extraction form were refined, expanded, and merged as needed. Members of the core coder team (AM, AY, LF, JT) then applied the updated data extraction form to 10% of the incident reports (*n* = 22) to assess fit to the data and enhance inter-rater reliability. All 211 records were then stratified into four groups (Groups A–D), each containing approximately 53 incident reports. These four groups were then coded independently, and in duplicate, by members of the core coder team: Group A (coded by AM and JT), Group B (JT and LF), Group C (AY and LF), and Group D (AM and AY). Extracted data were then de-duplicated, and discrepancies in extracted data were resolved by two members of the core coder team: AM (Groups B and C) and LF (Groups A and D). This stratified, multi-stage approach to coding was designed and implemented to ensure that each record was reviewed in full by three separate members of the coding team. This study did not require oversight from an institutional review board because it involved the analysis of preexisting, deidentified data, and no members of the study team had access to subject identifiers.

### Key variables

The primary outcome of interest in this analysis was whether police administered naloxone when responding to an overdose (categorized: yes, no, unclear). This information was extracted from the narrative section of each incident report.

We selected a range of incident characteristics that were hypothesized to influence whether police administered naloxone when responding to the incident. These included the racial/ethnic composition of the neighborhood of the incident (predominantly Latinx/Black, predominantly white, ZIP code redacted); ZIP codes in which greater than 50% of residents identified as Latinx or Black were classified as “predominantly Latinx/Black,” whereas ZIP codes in which greater than 50% of residents identified as white were classified as “predominantly white.” ZIP code-level race and ethnicity data were derived from the 2020 Census State Redistricting Data Summary File [35]. We extracted the month of the incident (six months pre-COVID vs. six months during COVID); day of the week (each day; Sunday through Saturday); time of day (morning, 5:00–11:59 am; afternoon, 12:00–4:59 pm; evening, 5:00–8:59 pm; night, 9:00 pm–4:59 am); whether an arrest was made (yes vs. no); whether property was seized (yes vs. no); whether drugs were seized (yes vs. no); the number of persons from whom police collected information on scene (i.e., number of subjects); the number of persons who overdosed (one vs. two or more); whether the Fire Department Rescue division (hereafter “Rescue”) was present before police arrival (yes, no, unclear); whether naloxone was administered by others, including Rescue (yes, no, unclear); others who administered naloxone (Rescue, bystander, both Rescue and bystander); timing of naloxone administration by others (before police arrival, while police present, both before police arrival and while police present, unclear); whether there were any references to fentanyl in the narrative (yes vs. no); whether there was any reference to a suicide attempt in the narrative (yes vs. no); whether the person who overdosed was transported to a hospital (yes, no, unclear); whether the overdose was described as fatal at the scene (yes vs. no); and the incident setting (private, public, vehicle, other, unclear).

We also selected several individual characteristics of people who overdosed that were hypothesized to influence whether police administered naloxone when responding to the incident. These included race (Black, white, other, not reported or unknown; ethnicity was not available); sex (male, female, not reported); age; and whether the person was arrested (yes, no).

### Statistical analyses

Characteristics of incidents in which police responded to an overdose are presented for the overall sample and stratified by whether police administered naloxone (yes vs. no). Individual characteristics of people who overdosed are also reported for the overall sample and stratified by whether police administered naloxone. Bivariate associations were examined using Wilcoxon rank sum tests for continuous variables and Fisher’s exact tests for categorical variables. Fisher’s exact tests were selected for categorical variables because this statistical approach provides a conservative and reliable test of statistical significance when individual observations are independent and sample sizes are small [36, 37]. Descriptively, we also examined the amount of naloxone that police administered on scene, incidents in which arrests occurred, and incidents in which the person who overdosed was described as combative.

## Results

Among 211 incidents included in this analysis, we found that police administered naloxone in 10% (*n* = 21) of incidents. As detailed in Table1, most incidents (*n* = 141, 67%) occurred in predominantly Latinx/Black neighborhoods, and more than half of incidents (*n* = 123, 58%) in which police responded to an overdose occurred in the six months prior to the COVID-19 pandemic as compared to the subsequent six months beginning in March 2020. Police most frequently responded to overdoses on Tuesdays (*n* = 36, 17%), Thursdays (*n* = 33, 16%), and Saturdays (*n* = 31, 15%), and in the night (*n* = 75, 36%) or evening (*n* = 63, 30%). Arrests were made in a minority of incidents (*n* = 3, 1%). Property was seized in 15 incidents (7%), and among these, drugs or paraphernalia were seized in nine incidents. The median number of persons from whom police collected contact and demographic information (i.e., name, address, phone number, race, sex, date of birth or age) on scene was one (interquartile range [IQR]: 1, 2), although some incident reports contained up to five people. In most incidents (*n* = 202, 96%), there was only one person who overdosed, although nine incidents (4%) included two or more people who had overdosed.

**Table 1:** Characteristics of 211 incidents in which police responded to people who overdosed, stratified by whether police administered naloxone in a city in Rhode Island from September 1, 2019, to August 31, 2020

Characteristic		Overall (N = 211)	Police did not administer naloxone^a^ (*n* = 190)	Police administered naloxone (*n* = 21)	*P *value
Neighborhood of incident	Predominantly Latinx/Black	141 (67%)	129 (68%)	12 (57%)	0.13
	Predominantly white	68 (32%)	60 (32%)	8 (38%)	
	ZIP code redacted	2 (1%)	1 (1%)	1 (5%)	
Month of incident	Six months pre-COVID	123 (58%)	109 (57%)	14 (67%)	0.41
	Six months during COVID	88 (42%)	81 (43%)	7 (33%)	
Day of week	Sunday	29 (14%)	24 (13%)	5 (24%)	0.23
	Monday	24 (11%)	20 (11%)	4 (19%)	
	Tuesday	36 (17%)	34 (18%)	2 (10%)	
	Wednesday	28 (13%)	27 (14%)	1 (5%)	
	Thursday	33 (16%)	30 (16%)	3 (14%)	
	Friday	30 (14%)	25 (13%)	5 (24%)	
	Saturday	31 (15%)	30 (16%)	1 (5%)	
Time of day	Morning (5:00–11:59am)	23 (11%)	20 (11%)	3 (14%)	0.84
	Afternoon (12:00–4:59 pm)	50 (24%)	45 (24%)	5 (24%)	
	Evening (5:00–8:59 pm)	63 (30%)	56 (29%)	7 (33%)	
	Night (9:00 pm-4:59am)	75 (36%)	69 (36%)	6 (29%)	
Arrest made	Yes	3 (1%)	1 (1%)	2 (10%)	–
Property seized	Yes	15 (7%)	13 (7%)	2 (10%)	0.65
Drugs or paraphernalia seized	Yes	9 (4%)	8 (4%)	1 (5%)	> 0.99
Number of people^b^	Median (IQR)	1 (1, 2)	1 (1, 2)	1 (1, 2)	0.88
Number of people who overdosed	One	202 (96%)	182 (96%)	20 (95%)	> 0.99
	Two or more^c^	9 (4%)	8 (4%)	1 (5%)	
Rescue present before police arrival	Yes	124 (59%)	124 (65%)	0 (0%)	< 0.01
	No	59 (28%)	38 (20%)	21 (100%)	
	Unclear	28 (13%)	28 (15%)	0 (0%)	
Naloxone administered by others	Yes	138 (65%)	133 (70%)	5 (24%)	< 0.01
	No	45 (21%)	31 (16%)	14 (67%)	
	Unclear	28 (13%)	26 (14%)	2 (10%)	
Others who administered naloxone (*n* = 138)	Rescue	111 (80%)	106 (80%)	5 (100%)	> 0.99
	Bystander	17 (12%)	17 (13%)	0 (0%)	
	Rescue and bystander	10 (7%)	10 (78%)	0 (0%)	
Timing of naloxone administration by non-police (*n* = 138)	Before police arrival	41 (30%)	41 (31%)	0 (0%)	0.48
	While police present	85 (62%)	80 (60%)	5 (100%)	
	Both before police arrival and while police present	6 (4%)	6 (5%)	0 (0%)	
	Unclear	6 (4%)	6 (5%)	0 (0%)	
Reference to fentanyl	Yes	24 (11%)	22 (12%)	2 (10%)	> 0.99
Reference to suicide attempt	Yes	8 (4%)	8 (4%)	0 (0%)	> 0.99
Transported to hospital	Yes	199 (94%)	178 (94%)	21 (100%)	> 0.99
	No^d^	7 (3%)	7 (4%)	0 (0%)	
	Unclear	5 (2%)	5 (3%)	0 (0%)	
Overdose was fatal	Yes	6 (3%)	6 (3%)	0 (0%)	> 0.99
Incident setting	Private (e.g., someone’s home)	73 (35%)	67 (35%)	6 (29%)	< 0.01
	Public (e.g., store, outdoors)	53 (25%)	45 (24%)	8 (38%)	
	Vehicle	20 (10%)	13 (7%)	7 (33%)	
	Other^e^	8 (4%)	8 (4%)	0 (0%)	
	Unclear	57 (27%)	57 (30%)	0 (0%)	

^a^There were two records for which details were insufficient to determine whether police administered naloxone; for analytic purposes, there records were categorized as “police did not administer naloxone.”

^b^Refers to the number of persons from whom police collected information on scene. Information collected included name, address (where available), phone number, race, sex, and DOB/age

^c^Among records with two or more people who overdosed, 8 (89%) had two people who overdosed and 1 (11%) had three

^d^Three records noted that the subject refused transport to a hospital

^e^Other locations included homeless shelters, a college dorm room, and hotel rooms

Rescue was present before police arrival in most incidents (*n* = 124, 59%), and naloxone was most often administered by people other than police (*n* = 138, 65%). Naloxone was most frequently administered by Rescue (*n* = 111, 80%), bystanders (*n* = 17, 12%), or by both Rescue and bystanders (*n* = 10, 7%) among incidents in which individuals other than police administered it (*n* = 138). Naloxone was most frequently administered by others while police were present (*n* = 85, 62%), followed by before police arrival (*n* = 41, 30%).

Several incident reports reference fentanyl in the narrative (*n* = 24, 11%) or that the overdose was a suicide attempt (*n* = 8, 4%). In almost all cases, the person who overdosed was transported to the hospital (*n* = 199, 94%), and the overdose was described as fatal at the scene in six incidents (3%). Overdose incidents most frequently occurred in private settings (e.g., someone’s home; *n* = 73, 35%), followed by public (e.g., a store or outdoors; *n* = 53, 25%) and in a vehicle (*n* = 20, 10%); however, the incident setting could not be derived from approximately a quarter of incident reports (*n* = 57, 27%).

Characteristics of incidents in which police responded to an overdose, stratified by whether police administered naloxone, are presented in Table 1. In bivariate analyses, we found that police were significantly more likely to administer naloxone when they were first on the scene, when naloxone had not been previously administered by others, and when the overdoses occurred in public or in a vehicle. In the 21 incident reports where police administered naloxone, the amount of naloxone that was administered varied from four to 12 mg (i.e., one to four doses); reports also described “two administrations” and “two sprays” of naloxone.

Among the 211 incidents examined, there were 221 people who overdosed. However, there were seven incident reports (3%) in which demographic information of the person who overdosed could not be extracted due to over-redaction; thus, these records were excluded from analyses examining individual characteristics of people who overdosed (Table 2). We found that most people who overdosed were white (*n* = 157, 73%) and male (*n* = 148, 69%), and the median age was 34 (IQR: 27, 44). Among all people who overdosed, one (1%) was arrested. In bivariate analyses, we found that police were more likely to administer naloxone when the person who overdosed was Black (see Table 2).

**Table 2:** Individual characteristics of 214 people who overdosed in a city in Rhode Island from September 1, 2019, to August 31, 2020^a^

Characteristic		Overall (*N* = 214)	Police did not administer naloxone (*n* = 193)	Police administered naloxone (*n* = 21)	*P *value
Race	Black	42 (20%)	34 (18%)	8 (38%)	0.05
	White	157 (73%)	146 (76%)	11 (52%)	
	Other^b^	3 (1%)	2 (1%)	1 (5%)	
	Not reported or Unknown	12 (6%)	11 (6%)	1 (5%)	
Sex	Male	148 (69%)	133 (69%)	15 (71%)	> 0.99
	Female	65 (30%)	59 (31%)	6 (29%)	
	Not reported	1 (1%)	1 (1%)	0 (0%)	
Age^c^	Median (IQR)	34 (27, 44)	34 [27, 45]	32 [26, 40]	0.63
Arrested	Yes	1 (1%)	0 (0%)	1 (5%)	0.10

^a^Incident reports in which demographic information of the person who overdosed could not be extracted (*n* = 7; 3%) were excluded from analyses. Specifically, there were 4 records in which police collected demographic information from multiple persons on scene, but due to over-redaction, it was unclear which person had overdosed. Police administered naloxone in one of these incidents. There were 3 additional records in which information was collected from one person on scene; however, it was unclear from the record narrative whether the individual from whom demographic information was collected was the person who overdosed. Police did not administer naloxone in these incidents. No persons in these excluded records were arrested

^b^Other includes persons racialized as Asian or Native American

^c^Age is reported for the person who overdosed in all but 10 records; the field for age was blank in 6 records, redacted in 2 records, and listed as “unknown” in 2 records

### Incidents in which arrests occurred

Arrests were made in a total of three incidents (1%), including one in which the person who overdosed was arrested. Two of the three incidents occurred in private or semi-private (e.g., a hotel room) settings, and one occurred in a vehicle. In one incident, the person who overdosed was arrested for an outstanding warrant, possession of prohibited weapons, and drug possession, among other charges. In the remaining two incidents, other persons on scene were arrested. In one of these incidents, the parents of an infant who overdosed were arrested, and investigators from the state Department of Children, Youth, and Families (DCYF) arrived on scene prior to police. In the other incident, a roommate of the person who overdosed was arrested for an outstanding warrant. Among the four persons who were arrested, ages ranged from 32 to 40; two were white men, one was a white woman, and one was a Black man.

### Incidents in which the person who overdosed was described as combative

In three incidents (1%) the person who overdosed was described as combative. Of these, one incident occurred in a private setting, one in a public setting, and one in a setting that could not be determined from the incident report narrative. In one incident, the person who overdosed was restrained by fire personnel and handcuffed to the ambulance stretcher by police; upon arrival at the hospital, police removed the handcuffs and the person who overdosed was restrained by hospital security. In the second incident, the person who overdosed was described as combative and experiencing hallucinations; this person was also handcuffed to the ambulance stretcher and police assisted with transport to the hospital. In the third incident, the person who overdosed was handcuffed and transported to the hospital. Among the three people who were described as combative, ages ranged from 26 to 40; one was a white man, one was a white woman, and one was a Black man. No incidents in which the overdose victim was described as combative resulted in arrest.

## Discussion

In this sample of 211 incident reports in which police responded to an overdose, we found that police infrequently administered naloxone, primarily because they were infrequently the first to arrive on scene. In most incidents, Rescue arrived on scene before police, naloxone was administered by Rescue or a bystander, and the person who overdosed was transported to a hospital. Police were significantly more likely to administer naloxone when they were the first emergency responders to arrive on scene, when naloxone had not been previously administered on scene, and when the overdose occurred in public or in a vehicle. Police were also significantly more likely to administer naloxone when the person who overdosed was Black; however, this is likely because the majority (67%) of overdose incidents in which police responded to an overdose occurred in predominantly Latinx/Black neighborhoods, which may be attributable to increased police surveillance in these communities [17]. This finding might also reflect recent and dramatic increases in overdose mortality among persons who are Black and those who are Latinx [38, 39]. We found that arrests were rare, although there was one incident in which the person who overdosed was arrested. We also found that people who overdosed were rarely described as combative, and in these instances, police handcuffed the person who overdosed for transport to a local hospital.

Existing literature regarding the relative timing of police and other first responder arrival on scene is mixed. In a qualitative study of police, Rescue, and emergency medical service providers, police in six New Hampshire counties were typically the last to arrive on scene, although they were also often the last to be dispatched [7]. Conversely, in a novel study of body-worn camera footage of incidents in which police responded to overdoses in Arizona, police were the first to arrive on scene in most (74%) incidents, although these incidents were restricted to those in which police administered naloxone [40]. Other research has found that police may be among the first to arrive on scene in rural settings, such as remote areas of Missouri [10]. We also found that the person who overdosed was transported to a hospital in the vast majority (94%) of cases. This finding is consistent with previous research examining police use of naloxone in a large urban police agency (location not reported), which found that 97% of people who overdosed were transported to a hospital voluntarily [15]. Additionally, recent data from nonfatal opioid overdoses attended by emergency medical services in Rhode Island indicate high levels of hospital transport (99%) [41]. Overall, given the importance of medical intervention following an overdose, the low prevalence of hospital transport refusal across settings is promising.

Evidence regarding the prevalence of arrests when police respond to overdoses is similarly mixed. Our finding that arrests were rare (1%) when police respond to overdoses is consistent with findings from Seattle, Washington; investigators found in a survey of police that officers rarely reported that arrests were made at the most recent overdose to which they responded, with 1% of people who overdosed and 1% of bystanders being arrested [8]. Our findings, however, were divergent from research examining police use of naloxone in a large urban police agency, which found that 18% of people who overdosed were arrested at incidents in which police administered naloxone [15]. Our findings were also incongruous with recent research investigating emergency medical service incidents and jail booking events in Indianapolis, Indiana, which found that 10% of nonfatal overdoses were followed by incarceration within six hours of the overdose incident [42]. The low prevalence of arrests when police respond to overdoses in the current study suggests that the local police department is broadly compliant with the current Good Samaritan Law in Rhode Island, which provides people who overdose and those who seek medical assistance for them with immunity from prosecution for the possession of controlled substances or drug paraphernalia, among other protections [31]. Nonetheless, discrepancies in the prevalence of arrests across jurisdictions within the USA underscore significant heterogeneity in standard operating procedures when police respond to overdoses, as well as differences in Good Samaritan Law protections across states. Prior research has also documented that there are racial/ethnic disparities in knowledge of Good Samaritan Laws [43, 44]. While procedures and protections are heterogeneous across the USA, policies that strengthen and enhance Good Samaritan Laws—and increase awareness of these laws in all communities—are needed to prevent arrests when police are dispatched to overdose events, particularly in settings that lack more comprehensive Good Samaritan Law protections. Further research is also needed to systematically investigate and monitor police compliance with existing Good Samaritan Laws across the country.

Furthermore, in the current study, we identified three incidents in which arrests were made when police responded to an overdose: one in which a roommate of the person who overdosed was arrested, one in which the parents of the child who overdosed were arrested, and one in which the person who overdosed was arrested. The latter two incidents represented extraordinary circumstances involving minors or multiple offenses, although pre-arrest diversion approaches with comprehensive behavioral health care services and referral to treatment may have been appropriate [45–47]. In the former incident, police ran a warrant search on the roommate of the person who overdosed who, as the incident report details, was attempting to render aid. While it is unclear whether running a warrant search of bystanders to an overdose is a standard operating procedure in the department, police must not be permitted to do so. Extensive prior literature has documented that fear of arrest [18–21], including for outstanding warrants [22–26], strongly deters bystanders from seeking emergency services in the event of an overdose. Current Good Samaritan Laws should be expanded to provide immunity from arrest for an outstanding warrant when the individual was encountered by police as a result of seeking medical assistance for an overdose, along with other protections [28]. In the interim, overdose education and naloxone distribution programs should continue to emphasize the importance of seeking emergency medical services in the event of an overdose, the limited protections of existing Good Samaritan Laws, and strategies to mitigate the risk of arrest when seeking care for others. Police may also benefit from recurring training on protections offered by existing Good Samaritan Laws. Given the increasing complexity of overdoses within the current overdose crisis which can require urgent medical assistance [38, 48], strategies that maximize the availability of emergency services in the event of an overdose—and minimize hesitancy in calling 911—must be aggressively pursued.

Our finding that people who overdosed were rarely (1%) described by police as combative is supported by prior literature. A review of 800 reports in which police and/or fire responded to opioid-related overdoses in Erie County, New York found that people who overdosed were described as combative after naloxone administration in only 2% of incidents [16]. Similarly, research examining police use of naloxone in a large urban police agency found that people who overdosed were described as combative in less than 1% of incidents [15]. This finding, however, is incongruous with officers’ perceived role at the scene of an overdose. A survey of police in Seattle, Washington found that 77% of officers believed that it was important for police to be present at the scene of an overdose to keep medical personnel safe [8]. This marked discrepancy in combativeness and officers’ perceived role of protecting medical personnel suggests that police may greatly overestimate the risks that bystanders and people who overdose present to others. This misconception may be corrected through additional training of police [49] or additional experience in responding to overdose incidents and administering naloxone [50].

Overall, our findings that [1] police were rarely needed to administer naloxone, [2] arrests were made for outstanding warrants in some incidents, and [3] people who overdose were rarely described as combative underscore that—ideally—all jurisdictions should have sufficient emergency medical service personnel to ensure a rapid response to overdose events, with police rarely or never dispatched to respond to overdoses. Given that fear of arrest is a strong and well-documented deterrent to seeking emergency services in the event of an overdose [18–26], ending the practice of routine police attendance at overdoses would increase calls for emergency medical services at overdose events and increase successful overdose reversals at a time when overdose fatalities have reached unprecedented heights. However, until this ideal can be achieved, any available first responders should be dispatched concurrently to minimize total response time and prevent overdose death. Police should be instructed to resume their patrol once other professional responders arrive on scene, and police should be prohibited from taking non-essential actions that would discourage future calls for assistance (e.g., running warrant searches of persons on scene). Additionally, when emergency medical services are the first to respond, police should be instructed by dispatch to resume their patrol given that their attendance is no longer required.

Critically, the incidents analyzed as part of this analysis represent a significant under-count of overdoses occurring in this city in Rhode Island. While the current analysis examines 211 incidents in which police responded to an overdose, there were approximately 480 emergency medical service runs for nonfatal opioid-related overdoses [51] and approximately 80 fatal overdoses [52] occurring during the same period in the city of focus. Furthermore, due to the specificity of the case definition used to identify suspected nonfatal opioid-related overdoses in emergency medical service data (i.e., the determination was based on primary and secondary impression, whether or not an overdose term was mentioned in the case narrative or chief complaint, and if naloxone was administered) and the exclusion of fatal overdoses, the number of emergency medical services runs for suspected opioid overdoses is also an undercount of the total number of overdoses that occur in the city, especially as approximately half of overdoses are self-managed without support from first responders [53–56].

Our findings should be interpreted in light of several limitations. First, the local Public Records Unit identified and released incident reports in which police responded to an overdose; the “incident type” (i.e., as noted in the case section of each incident report) of all records that were released and included in this analysis was “overdose.” However, the criteria for record inclusion and exclusion were not specified by this department, and it is not possible to confirm whether any incident reports were withheld or incorrectly excluded. Nonetheless, we expect that the agency has made a good faith attempt to capture and release all relevant reports. Second, non-identifiable information such as ZIP code and age could not be extracted from some incident reports due to over-redaction. While data missingness may bias study results, only a small portion of the total incident reports examined were affected by over-redaction, and we do not have reason to believe that over-redaction occurred systematically. Thus, we expect that data missingness is a minimal threat to the reliability and validity of current findings. Third, this analysis included all incident reports in which police responded to an overdose; thus, there were some incidents in which police administration of naloxone was not indicated, such as overdose incidents that were not opioid-related and incidents in which the person who overdosed was conscious or alert at police arrival. Finally, we examined incident reports in which officers of the local city police department responded to an overdose. Our findings may not be generalizable to other jurisdictions, including other cities in Rhode Island.

## Conclusions

In this retrospective analysis of incident reports in which police responded to an overdose, we found that police were rarely needed to administer naloxone, as naloxone was administered by Rescue or a bystander in most incidents analyzed, and police were the last to arrive on scene. While arrests were rare, in some incidents, arrests were made for outstanding warrants, suggesting a need for expanded Good Samaritan Law protections. We also found that people who overdosed were rarely described as combative by police, which challenges the role and utility of police attendance at overdose events. Taken together, our findings indicate that, ideally, all jurisdictions should have sufficient emergency medical service personnel to ensure a rapid response to overdose events, with police rarely or never dispatched to respond to overdoses. However, until this ideal can be achieved, any available responders should be dispatched concurrently, with police instructed to resume patrol once other professional responders arrive on scene. Moreover, police should be prohibited from taking non-essential actions that would discourage future calls for assistance, such as running warrant searches of persons on scene.

## Supplementary Information

**Additional file 1**. Appendix for the final data extraction form.

## Data Availability

The datasets that were used and analyzed as part of the present study are available from the corresponding author on reasonable request.
