# Supplementary material for: Characteristics of events in which police responded to overdoses: an examination of incident reports in Rhode Island
Source: Harm Reduct J. 2022 Oct 18;19:116. doi: 10.1186/s12954-022-00698-2 (PMC9578237; doi:10.1186/s12954-022-00698-2)
Supplement: Supplementary file 1 — Additional file 1. Appendix for the final data extraction form. [file 12954_2022_698_MOESM1_ESM.pdf]

Basic Info

Case number

Individual identifiers

Location zip

Zip code of location of incident. If zip code is redacted, please search for the zip code for the city/town and enter it here. If multiple zip codes exist for the city/town, please list the town name. If no city/town name is provided, please note "redacted"

Reporting officer ID

Please copy/paste number and surname

Reported date

Date incident occurred

Time of incident

- Morning (5-11:59)
- Afternoon (12-16:59)
- Evening (17-20:59)
- Night (21-4:59)

Day of week

- Sunday
- Monday

- Tuesday
- Wednesday
- Thursday
- Friday
- Saturday

Are offense details present?

Indicator for whether offenses are present

- Yes
- No
- Crime code 99/Statute not used

How many offenses are listed?

Indicator for the number of total offenses

Offense description(s)

If multiple offense descriptions are present, separate by "AND"

Number of subjects

Number of subjects involved in the incident (note: must be a number). DO NOT include "society" when counting subjects.

Subjects

Subject 1

Type (Subject 1)

Subject #1 type of person

- Other (reporting person)
- Suspect

- Victim
- Witness
- ____ Something else

Zip code (Subject 1)

Subject #1 zip code. If unavailable, write "redacted".

____

Race (Subject 1)

Subject #1 race

- White
- Black
- Asian
- Native American
- Other
- Unknown
- Not reported

Sex (Subject 1)

Subject #1 sex

- Male
- Female
- Unclear/not stated

Age (Subject 1)

Subject #1 age

____

Suspected overdose victim? (Subject 1)

Indicator for whether the police suspected that Subject #1 had overdosed

- Yes
- No

- ○ Unclear/not stated

Arrested? (Subject 1)

Indicator for whether Subject #1 was arrested

- ○ Yes
- ○ No
- ○ Unclear/not stated

Arrest type? (Subject 1)

Type of arrest of Subject #1

[ ]

Subject 2

Type (Subject 2)

Subject #2 type of person

- ○ Other (reporting person)
- ○ Suspect
- ○ Victim
- ○ Witness
- ○ [ ] Something else

Zip code (Subject 2)

Subject #2 zip code. If unavailable, write "redacted".

[ ]

Race (Subject 2)

Subject #2 race

- ○ White
- ○ Black
- ○ Asian
- ○ Native American

- Other
- Unknown
- Not reported

Sex (Subject 2)

Subject #2 sex

- Male
- Female
- Unclear/not stated

Age (Subject 2)

Subject #2 age

Suspected overdose victim? (Subject 2)

Indicator for whether the police suspected that Subject #2 had overdosed

- Yes
- No
- Unclear/not stated

Arrested? (Subject 2)

Indicator for whether Subject #2 was arrested

- Yes
- No
- Unclear/not stated

Arrest type? (Subject 2)

Type of arrest of Subject #2

Subject 3

Type (Subject 3)

Subject #3 type of person

- Other (reporting person)
- Suspect
- Victim
- Witness
- [ ] Something else

Zip code (Subject 3)

Subject #3 zip code. If unavailable, write "redacted".

Race (Subject 3)

Subject #3 race

- White
- Black
- Asian
- Native American
- Other
- Unknown
- Not reported

Sex (Subject 3)

Subject #3 sex

- Male
- Female
- Unclear/not stated

Age (Subject 3)

Subject #3 age

Suspected overdose victim? (Subject 3)

Indicator for whether the police suspected that Subject #3 had overdosed

- Yes
- No
- Unclear/not stated

Arrested? (Subject 3)

Indicator for whether Subject #3 was arrested

- Yes
- No
- Unclear/not stated

Arrest type? (Subject 3)

Type of arrest of Subject #3

Subject 4

Type (Subject 4)

Subject #4 type of person

- Other (reporting person)
- Suspect
- Victim
- Witness
- Something else

Zip code (Subject 4)

Subject #4 zip code. If unavailable, write "redacted".

Race (Subject 4)

Subject #4 race

- White
- Black
- Asian
- Native American
- Other
- Unknown
- Not reported

Sex (Subject 4)

Subject #4 sex

- Male
- Female
- Unclear/not stated

Age (Subject 4)

Subject #4 age

Suspected overdose victim? (Subject 4)

Indicator for whether the police suspected that Subject #4 had overdosed

- Yes
- No
- Unclear/not stated

Arrested? (Subject 4)

Indicator for whether Subject #4 was arrested

- Yes
- No
- Unclear/not stated

Arrest type? (Subject 4)

Type of arrest of Subject #4

[ ]

Subject 5

Type (Subject 5)

Subject #5 type of person

- ○ Other (reporting person)
- ○ Suspect
- ○ Victim
- ○ Witness
- ○ [ ] Something else

Zip code (Subject 5)

Subject #5 zip code. If unavailable, write "redacted".

[ ]

Race (Subject 5)

Subject #4 race

- ○ White
- ○ Black
- ○ Asian
- ○ Native American
- ○ Other
- ○ Unknown
- ○ Not reported

Sex (Subject 5)

Subject #4 sex

- ○ Male
- ○ Female
- ○ Unclear/not stated

Age (Subject 5)

Subject #5 age

Suspected overdose victim? (Subject 5)

Indicator for whether the police suspected that Subject #5 had overdosed

- Yes
- No
- Unclear/not stated

Arrested? (Subject 5)

Indicator for whether Subject #5 was arrested

- Yes
- No
- Unclear/not stated

Arrest type? (Subject 5)

Type of arrest of Subject #5

Arrest

Are arrest details present?

- Yes
- No

Number of people who were arrested?

Indicate the number of people who were arrested

Are property details present?

- Yes
- No

How many items were seized?

There will be one entry per unique item seized (entry must be a number)

Property code (Item 1)

Property type (Item 1)

Property description (Item 1)

If multiple property descriptions are present, separate by "AND"

Property code (Item 2)

Property type (Item 2)

Property description (Item 2)

If multiple property descriptions are present, separate by "AND"

Property code (Item 3)

Property type (Item 3)

Property description (Item 3)

If multiple property descriptions are present, separate by "AND"

Property code (Item 4)

Property type (Item 4)

Property description (Item 4)

If multiple property descriptions are present, separate by "AND"

Are chain of custody details present?

- Yes
- No

Are vehicle details present?

This refers only to the section with vehicle role, type, etc.

- Yes
- No

Vehicle role

Vehicle type

Finish

Number of suspected overdose victims?

Indicator for the number of people who overdosed

[ ]

Was Fire, EMS, or Rescue present before police arrival?

- ○ Yes
- ○ No
- ○ Unclear

Which was present before police arrival?

- ☐ Fire/Rescue
- ☐ EMS

Was naloxone administered by **non-police**?

- ○ Yes
- ○ No
- ○ Unclear

Does the record indicate that non-police were "working on" or otherwise responding to the victim?

- ○ Yes
- ○ No
- ○ Unclear

Who administered naloxone?

Indicator for who administered naloxone before police arrival

- ☐ Fire/EMS/Rescue
- ☐ Bystander (nonmedical)
- ☐ [ ] Other

Amount of naloxone administered by non-police (in doses or mg, specify)

When was naloxone administered by non-police?

- ☐ Before police arrival
- ☐ While police were present
- ☐ Unclear

Was naloxone administered by **police**?

- ○ Yes
- ○ No
- ○ Unclear

Amount of naloxone administered by police (in doses or mg, specify)

Any reference to the subject being alert or responsive?

If multiple overdose victims, indicate whether at least one was alert or responsive.

- ○ Yes
- ○ No

Any reference to fentanyl or "possible fentanyl" use?

- ○ Yes
- ○ No

Transported to a hospital

Indicator for whether the overdose victim was transported to hospital

- ○ Yes
- ○ No
- ○ No - refused to be taken
- ○ Unclear

Does the record mention that the overdose was fatal?

- Yes
- No
- Unclear

Does the record mention that the overdose was a suicide attempt?

- Yes
- No
- Unclear

In what **type** of location did the event occur?

- Public (e.g., store, outdoors)
- Private (e.g., someone's home)
- [ ] Other
- Unclear

Was the overdose victim already deceased at police arrival? (i.e., dead on arrival)

- Yes
- No
- Unclear

Copy/paste the narrative

If unable to copy/paste, please indicate "unable to copy/paste" and skip

Notable notes

Notable aspects from the case

Flag to discuss further

- Yes
- No

Coder initials

Powered by Qualtrics
